# Point-of-care molecular diagnostics and drug-resistance mechanisms in neglected infectious diseases: current advances and future therapeutic opportunities

**DOI:** 10.3389/fcimb.2026.1769679

**Published:** 2026-02-26

**Authors:** Qi Zhang, Xiajun Zhang, Jie Yang, Hongliang Li

**Affiliations:** 1 Department Clinical Laboratory, The People’s Hospital of Danyang, Danyang Hospital Affiliated to Nantong University, Jiangsu, China; 2 Department Infectious Diseases, The People’s Hospital of Danyang, Danyang Hospital Affiliated to Nantong University, Jiangsu, China

**Keywords:** antimicrobial resistance (AMR), biosensors and microfluidic platforms, isothermal amplification and CRISPR-based assays, molecular detection technologies, point-of-care diagnostics

## Abstract

The rising burden of neglected infectious diseases and the accelerating spread of antimicrobial resistance (AMR) demand rapid, accurate, and decentralized diagnostic solutions. Point-of-care (POC) molecular diagnostics enable early diagnosis and resistance profiling during the clinical encounter, without reliance on centralized laboratories, which is particularly important in low-resource settings. Molecular POC technologies are being developed around the following advances: isothermal nucleic acid amplification technologies, rapid polymerase chain reaction (PCR), CRISPR-based diagnostic detection technologies, nanomaterials-enabled biosensors, and microfluidic platforms for sample-to-results with near laboratory-quality accuracy within clinically relevant timeframes (typically < 30 minutes). The combination of artificial intelligence (AI) and cloud-based digital health systems supports the automated interpretation and provision of real-time surveillance and antimicrobial stewardship. Next-generation molecular POC platforms provide higher sensitivity and mechanistic insights into drug-resistance in TB, malaria, and bacterial infections. Barriers include clinical validation, cost, scalability, and equitable access. The convergence of molecular diagnostics, nanotechnology and AI-powered analytics leads to future-oriented, transformative opportunities in precision therapy areas, AMR surveillance and infectious disease preparedness.

## 1 Introduction

Infectious diseases remain a dominant cause of global morbidity and mortality, with a disproportionate burden borne by low- and middle-income countries (LMICs) where neglected infectious diseases such as tuberculosis, parasitic infections, sexually transmitted infections, and drug-resistant bacterial sepsis persist. Delays in accurate diagnosis in LMICs can lead to inappropriate antimicrobial usage and ongoing transmission of disease, leading to the accelerated evolution of antimicrobial resistance (AMR) (Madaje et al., 2025). An estimated 4.95 million deaths occur annually due to AMR, and 1.27 million deaths occur annually from drug resistance infection (Murray et al., 2022). There is an urgent need for faster diagnostics that are actionable during the patient’s first interaction with a healthcare provider, allowing immediate and appropriate antimicrobial therapy to be initiated. Point-of-care (POC) diagnostics have been established as a key component in the management of modern-day infectious diseases. POC diagnostics allow distributed testing, shortened turnaround times for diagnosis, and immediate treatment decisions, all of which are critical in locations that lack laboratory resources and infrastructure (Kardjadj, 2025).

Over the past decade, technological advances have substantially expanded the capabilities of POC diagnostics. Immunoassays are still the predominant type of POC test used, as they are cheap and simple to use, but tend to have very low sensitivity levels for when a person may be infected but is still in the early stages of infection or a person who has a low viral or bacterial burden. Molecular POC platforms, including isothermal amplification (LAMP, RPA), rapid PCR, CRISPR-based assays, and integrated sample-to-answer cartridges, can deliver clinically actionable results within approximately 15–60 minutes, depending on workflow and specimen type (Kinnamon et al., 2022). Advances in nanomaterial-enabled biosensors, graphene-based transducers, plasmonic nanoparticles, and paper-based microfluidic devices have improved analytical sensitivity, although reported limits of detection are often derived from controlled or contrived matrices and may not fully translate to complex clinical samples. The global market for infectious disease POC diagnostics continues to expand rapidly, and is continuing to expand very quickly, mainly due to the rapid escalation of AMR, the need to prepare for future pandemics, and the increased use of these technologies in both primary and decentralized settings (Naghavi et al., 2024).

Digital health technologies integrated into POC molecular diagnostics have proven equally transformative in supporting automated quality control, standardizing result interpretation and enabling nearly real-time AMR surveillance by linking clinical diagnostics with public health intelligence through the use of smartphone-connected reading devices, cloud-based data platforms and artificial intelligence (AI) to provide user-friendly technology that is transforming the way infectious disease is diagnosed and treated (Kardjadj, 2025). Such integration is particularly beneficial for neglected infectious diseases, as the under-reporting of AMR patterns represents one of the greatest challenges to addressing the public health impact of these diseases. Concurrently with these rapid developments in technology and their impact on patient care and treatment, expectations from regulatory authorities have changed substantially (Sharma et al., 2025). The U.S. Food and Drug Administration (FDA), the European Union *In Vitro* Diagnostic Regulation (IVDR) and the World Health Organization (WHO) have all issued guidance regarding the need for lifecycle-based validation, human factor engineering and continued performance monitoring following the launch of a product. Collectively, the convergence of molecular diagnostics, nanotechnology, AI-based data analytics and resistance-directed testing will redefine the way infectious disease care is delivered, allowing greater opportunities for personalized therapy, enhancing stewardship and presenting new avenues to combat drug-resistant neglected infectious diseases (Kahles et al., 2025; Kardjadj, 2025).

## 2 Drug-resistance in infectious diseases: challenges & need for POC resistance diagnostics

### 2.1 The public health threat of AMR & limitations of conventional diagnostics

**Figure 1:**
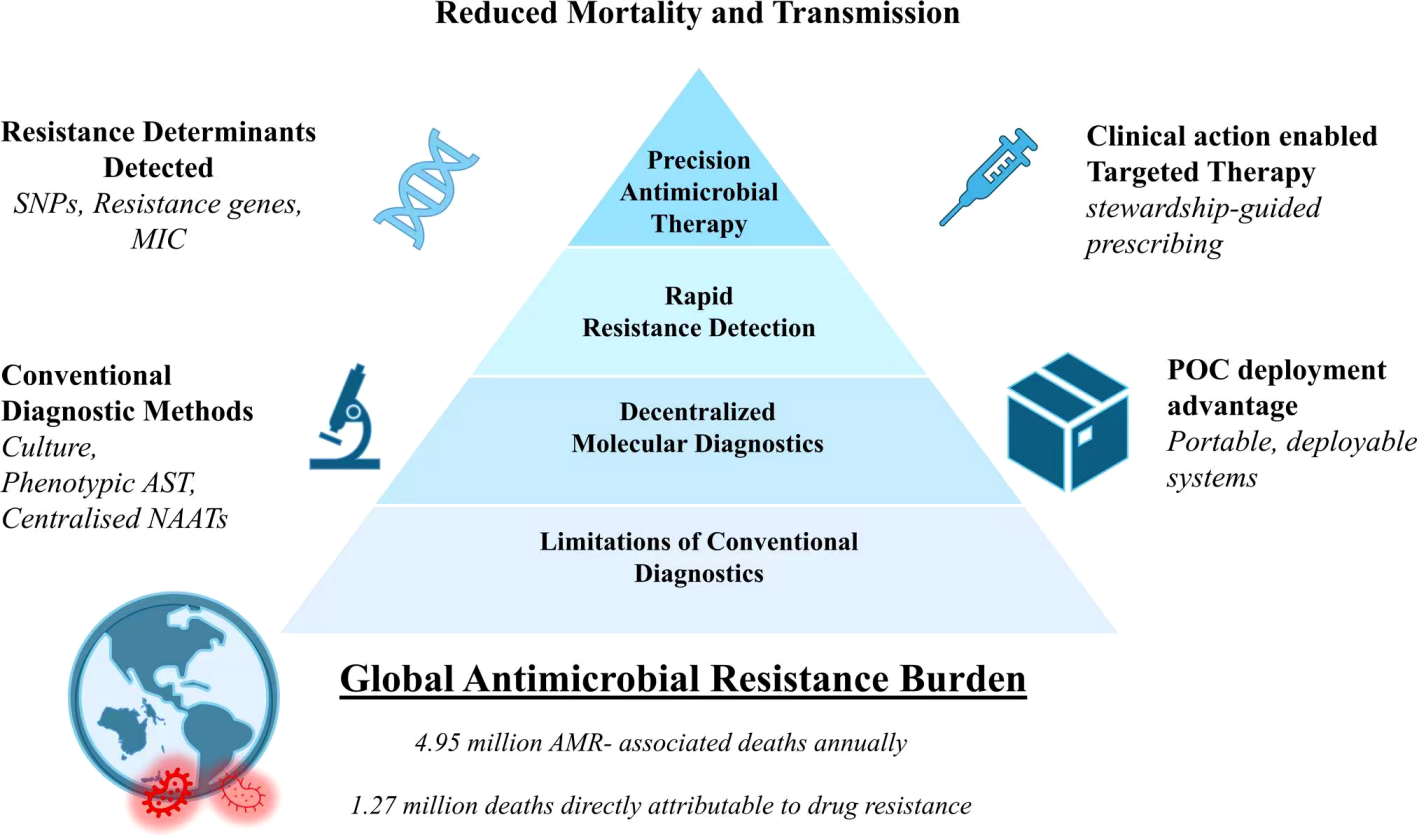
Global burden of antimicrobial resistance and conceptual value of point-of-care resistance diagnostics (POC-RD). AMR, Antimicrobial resistance; POC, Point-of-care; AST, Antimicrobial susceptibility testing; NAATs, Nucleic acid amplification tests; SNPs, Single-nucleotide polymorphisms; MIC, Minimum inhibitory concentration.

AMR represents one of the most pressing global public health crises of the 21st century. The burden of AMR is particularly severe for neglected and poverty-associated infectious diseases, where delayed diagnosis, limited access to susceptibility testing, and fragmented healthcare infrastructures exacerbate treatment failure and transmission. Recent estimates indicate that AMR was associated with approximately 4.95 million deaths worldwide, with 1.27 million deaths directly attributable to infections caused by drug-resistant pathogens (Murray et al., 2022). A major contributor to this crisis is the continued reliance on conventional diagnostic workflows that fail to deliver timely and actionable information for clinical decision-making. Traditional diagnostic approaches including culture-based identification, phenotypic antimicrobial susceptibility testing (AST), and centralized nucleic acid amplification tests (NAATs) remain the clinical gold standard but are intrinsically constrained by prolonged turnaround times, typically ranging from 24 to 96 hours. They also demand specialized infrastructure and trained personnel, making them impractical for urgent or remote clinical scenarios (Walker et al., 2025). As a result, clinicians frequently initiate empirical broad-spectrum antimicrobial therapy, which, while life-saving in some contexts, significantly accelerates selective pressure and the emergence of resistant strains. Diagnostic uncertainty thus drives inappropriate prescribing and further spreads resistance. The impact of these diagnostic delays is particularly evident for high-burden pathogens such as *Mycobacterium tuberculosis*, *Klebsiella pneumoniae*, *Pseudomonas aeruginosa*, *Neisseria gonorrhoeae*, and *Plasmodium falciparum*. These organisms are increasingly associated with multidrug-resistant (MDR), extensively drug-resistant (XDR), and, in rare but alarming cases, pan-drug-resistant phenotypes, complicating treatment and increasing morbidity and mortality, as illustrated in **Figure 1** (Alatawi et al., 2025; Roy et al., 2025).

### 2.2 Conceptual value of POC resistance diagnostics

**Figure 2:**
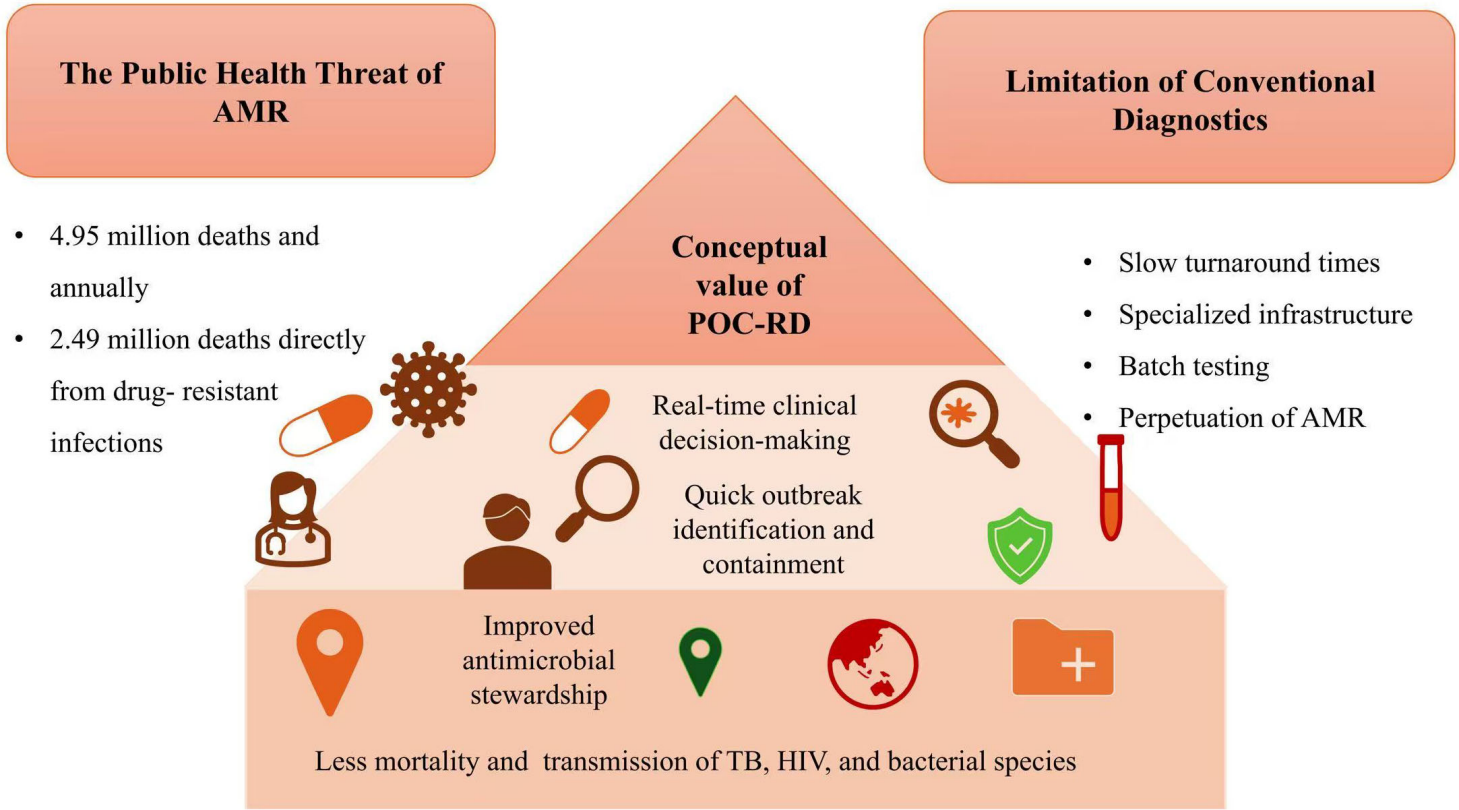
Conceptual value of point-of-care resistance diagnostics (POC-RD). Human Immunodeficiency Virus (HIV) is included specifically in the context of antiretroviral drug resistance monitoring in decentralized care settings; TB, Tuberculosis; AMR, Antimicrobial resistance; bacterial pathogens” refers to clinically relevant drug-resistant organisms, including *Escherichia coli*, *Klebsiella pneumoniae*, and *Staphylococcus aureus*.

Point-of-care resistance diagnostics (POC-RD) fundamentally redefine how antimicrobial resistance is detected, interpreted, and acted upon by compressing the diagnostic therapeutic decision loop to the earliest stage of clinical contact. Unlike conventional laboratory workflows, which often generate resistance data only after empirical treatment has already been initiated, point-of-care resistance diagnostics (POC-RD) are designed to detect clinically actionable resistance determinants in near real time. These include *rpoB* and *katG* mutations in *Mycobacterium tuberculosis*; fluoroquinolone-associated *gyrA* variants; carbapenemase genes such as *bla*_NDM_ and *bla*_KPC_; macrolide resistance mediated by *erm* genes; and molecular markers of antimalarial drug resistance. Depending on the diagnostic workflow and specimen type, results can be delivered within clinically relevant timeframes ranging from under one hour to several hours, enabling earlier optimization of therapy and infection control interventions. As illustrated in **Figure 2**, timely resistance-guided treatment facilitated by POC-RD contributes to reduced mortality and transmission of drug-resistant tuberculosis, HIV (antiretroviral resistance), and priority bacterial pathogens such as *Escherichia coli*, *Klebsiella pneumoniae*, and *Staphylococcus aureus* (Mani et al., 2001; Singh et al., 2021). By providing early resistance information, POC-RD allows clinicians to select the most effective therapy from the outset, reducing treatment failure and unnecessary drug exposure. The principal clinical value of POC-RD lies in its ability to support evidence-based prescribing decisions earlier in the care pathway. Early access to resistance information allows clinicians to select the most effective antimicrobial or direct-acting antiviral regimens from the outset, reducing treatment failure, shortening time to clinical stabilization, and limiting unnecessary drug exposure. In severe infections such as sepsis, drug-resistant tuberculosis, and invasive hospital-acquired infections, even short delays in appropriate therapy are associated with increased mortality and prolonged hospitalization (Oliveira et al., 1920). In severe infections such as sepsis, drug-resistant tuberculosis, and invasive hospital-acquired infections, delays in appropriate therapy are associated with increased mortality and prolonged hospitalization; earlier access to resistance information may therefore contribute to improved outcomes in selected high-risk contexts. By providing actionable resistance profiles in real time, POC-RD mitigates these risks and aligns clinical practice with evidence-based, pathogen-directed treatment principles (Kumar et al., 2024). At the population level, POC-RD plays a critical role in strengthening antimicrobial stewardship by curbing the routine use of broad-spectrum agents and preserving last-line therapies. By confirming resistance mechanisms on site, POC-RD enables rational de-escalation and limits the spread of resistant strains. This function is especially valuable in high-burden healthcare environments, where empirical prescribing is often driven by diagnostic uncertainty rather than microbiological evidence (Chigozie et al., 2025). Beyond individual patient management, resistance-aware POC diagnostics generate high-resolution, temporally relevant data that can bridge critical gaps in AMR surveillance, particularly in regions where laboratory reporting is sparse or delayed. POC-RD also offers substantial advantages for outbreak detection and containment. Portable and deployable resistance diagnostic systems enable rapid screening in hospitals, community settings, border regions, and field environments, facilitating early identification of resistant strains before widespread transmission occurs. Connected POC-RD devices can feed real-time data into decentralized surveillance networks, supporting local, regional, and national AMR monitoring (Kost, 2019). This capability is particularly important in low-resource and remote settings, where access to centralized laboratories is limited and resistance data are frequently underreported. Overall, the integration of POC-RD into clinical and public health workflows offers a promising pathway to earlier resistance awareness, improved stewardship practices, and enhanced surveillance, particularly for high-impact infectious diseases such as tuberculosis, HIV-associated infections, and bacterial sepsis (Heidt et al., 2020).

## 3 Current status: POC molecular platforms with drug-resistance detection

Rapid advances in molecular biology, microengineering, and digital health have enabled the emergence of point-of-care (POC) molecular diagnostic platforms capable of detecting both infectious agents and their associated drug-resistance determinants within clinically actionable timeframes. Unlike traditional centralized diagnostics, which are constrained by long turnaround times and infrastructure dependence, modern POC molecular systems offer integrated, sample-to-answer workflows that support real-time therapeutic decision-making (Zu et al., 2025). They are especially relevant for neglected infectious diseases such as tuberculosis, malaria, leishmaniasis, and multidrug-resistant bacterial infections, where rapid detection of resistance can guide targeted therapy and limit transmission. Here, we summarize the main classes of POC molecular diagnostics, their technical principles, clinical utility, and limitations (Liu et al., 2023).

### 3.1 Cartridge-based NAATs: Xpert MTB/XDR for tuberculosis

Cartridge-based nucleic acid amplification tests (NAATs) represent the most mature and widely deployed class of POC molecular diagnostics with integrated resistance detection. Among these, the Xpert MTB/XDR platform exemplifies how automated real-time PCR technology can be translated into robust, near-patient testing solutions for drug-resistant tuberculosis (TB) (Sahana et al., 2017). These systems employ fully enclosed, disposable cartridges that integrate sample preparation, nucleic acid extraction, amplification, and detection into a single workflow, minimizing hands-on time and biosafety risks. Following minimal sample processing, sputum specimens are loaded into cartridges that detect *Mycobacterium tuberculosis* complex DNA while simultaneously interrogating mutations in key resistance-associated genes, including *rpoB* (rifampicin resistance), *katG* and *inhA* (isoniazid resistance), and *gyrA*/*gyrB* (fluoroquinolone resistance) (Rozales et al., 2013; Park et al., 2018). The clinical impact of cartridge-based NAATs is largely driven by their rapid turnaround time, typically under two hours, which contrasts sharply with conventional culture-based diagnostics and phenotypic drug susceptibility testing (DST) that may require weeks to generate actionable results. Numerous multicenter evaluations have demonstrated high concordance between Xpert MTB/XDR results and gold-standard phenotypic DST or sequencing-based reference methods, with reported sensitivities and specificities frequently exceeding 90% for major resistance determinants (Anton-Vazquez et al., 2021). Early resistance detection facilitates earlier treatment optimization, supports transmission control efforts, and contributes to resistance surveillance. Beyond individual patient management, the public health value of these platforms lies in their capacity to rapidly identify drug-resistant TB at first presentation, thereby reducing delays to effective therapy, limiting onward transmission, and supporting surveillance of resistant TB strains (Liebenberg et al., 2022). However, limitations remain, including dependence on stable electricity, relatively high per-test costs, and restricted ability to detect novel or uncommon resistance mutations outside predefined assay targets. Nevertheless, cartridge-based NAATs continue to serve as a benchmark for POC resistance diagnostics and illustrate the feasibility of molecular DST outside centralized laboratories (Chakraborty, 2024).

### 3.2 CRISPR-based point-of-care assays

CRISPR-based diagnostic technologies represent a rapidly evolving frontier in POC molecular testing, offering unprecedented specificity, adaptability, and speed. Platforms built on CRISPR-Cas12 and Cas13 systems exploit the collateral cleavage activity triggered upon sequence-specific recognition of target DNA or RNA. Once activated, Cas enzymes cleave labeled reporter molecules, generating detectable fluorescence, colorimetric, or electrochemical signals. These assays typically operate under isothermal conditions, eliminating the need for thermal cycling and enabling simplified instrumentation suitable for decentralized settings (Ghorbani et al., 2021). CRISPR-based POC assays have demonstrated the ability to detect pathogen-specific nucleic acids as well as single-nucleotide polymorphisms (SNPs) associated with antimicrobial resistance. Proof-of-concept studies have validated their capacity to discriminate resistance-conferring mutations such as gyrA substitutions associated with fluoroquinolone resistance and carbapenemase genes including *bla*_NDM_ and *bla*_KPC_ with high analytical specificity (Yang et al., 2023). SNP-level detection is critical for accurate resistance profiling, and programmable guide RNAs allow rapid adaptation to emerging resistance mechanisms in neglected diseases such as malaria or MDR bacterial infections (Kohabir et al., 2025; Zhuang et al., 2025). Moreover, assay times typically range from 20 to 40 minutes, supporting near-real-time clinical decision-making as some CRISPR assays are performed on benchtop instruments in high-complexity laboratories, others are adapted for decentralized near-patient testing with minimal equipment, while true point-of-care systems integrate lateral flow or microfluidic cartridges for rapid, on-site results without specialized infrastructure. Time-to-result and operational requirements vary substantially across these formats. These assays hold high potential for next-generation POC diagnostics but require further clinical validation and regulatory approval. Regulatory approval pathways for CRISPR diagnostics are still evolving; for example, the FDA provides Emergency Use Authorizations (EUA) for rapid deployment, the EU IVDR framework establishes risk-based validation requirements, and the WHO prequalification program guides global adoption of high-priority diagnostics. Real-world performance data across diverse healthcare settings also remain limited (Wang et al., 2025; Zhou et al., 2026).

### 3.3 Biosensor-integrated systems (electrochemical, optical, nanomaterial-based)

Biosensor-integrated POC systems represent a complementary approach to nucleic acid amplification–based diagnostics, leveraging advances in nanotechnology, surface chemistry, and signal transduction. These platforms utilize molecular recognition elements—such as oligonucleotide probes, aptamers, or antibodies—coupled with nanomaterials including graphene, gold nanoparticles, and quantum dots to detect nucleic acids or resistance-associated proteins with exceptional sensitivity. Several research prototypes have reported detection limits in the femtomolar to picomolar range, enabling reliable analysis from very small sample volumes such as fingerstick blood, urine, or swab lysates (Chen et al., 2025). Electrochemical biosensors measure changes in current, voltage, or impedance upon target binding, while optical biosensors exploit fluorescence, plasmon resonance, or photonic signal changes. Nanomaterials enhance signal amplification, surface area, and binding kinetics, contributing to rapid assay times—often under 15 minutes—and low power requirements. In principle, these characteristics suggest suitability for decentralized testing and outbreak response; however, translating laboratory performance into consistent field-level reliability remains a major challenge (Grieshaber et al., 2008). These platforms are well-suited for field deployment in remote or resource-limited settings, crucial for neglected infectious diseases. In the context of AMR, biosensor-based systems have been explored for detecting resistance genes, enzymatic activity (e.g., β-lactamase function), and specific resistance-associated proteins. Their rapid turnaround and portability offer significant advantages; however, the translation of biosensor prototypes into clinically validated products is hindered by substantial challenges, including batch-to-batch reproducibility, matrix effects in complex clinical samples, long-term stability, limited multiplexing capacity, and a lack of standardized validation frameworks. At present, biosensor-integrated systems are best viewed as experimental or early-stage translational technologies, with strong long-term potential for POC resistance diagnostics but insufficient evidence to support routine clinical adoption at scale (Kao and Alocilja, 2025; Ait Lahcen et al., 2026).

### 3.4 Multiplex POC panels for broad infection/resistance screening

Multiplex POC molecular platforms represent a significant advance in syndromic diagnostics by enabling simultaneous detection of multiple pathogens and resistance determinants from a single sample. These systems employ multiplex PCR or microarray-based technologies to identify a broad spectrum of viruses, bacteria, and fungi alongside key antimicrobial resistance genes such as *mec*A, *bla*_NDM_, *bla*_KPC_, and *van*A/B. This comprehensive diagnostic approach is particularly valuable in clinical syndromes such as sepsis, respiratory tract infections, and sexually transmitted infections, where clinical presentation alone is insufficient to guide targeted therapy (Serapide et al., 2025). These panels provide pathogen ID and resistance profiles in 45–90 minutes, enabling rapid, evidence-based therapy and minimizing unnecessary antimicrobial use (Candel et al., 2024). Limitations include predefined panels and assay complexity, but multiplex POC systems remain powerful tools for high-priority neglected pathogens such as MDR TB, drug-resistant *N. gonorrhoeae*, and resistant malaria. Nonetheless, for high-priority pathogens such as methicillin-resistant *Staphylococcus aureus* (MRSA), carbapenem-resistant *Enterobacterales*, drug-resistant *Neisseria gonorrhoeae*, and multidrug-resistant TB, multiplex POC platforms offer a powerful diagnostic solution that bridges clinical microbiology and real-time therapeutic decision-making (Yamin et al., 2023).

### 3.5 Clinical and public health implications

**Figure 3:**
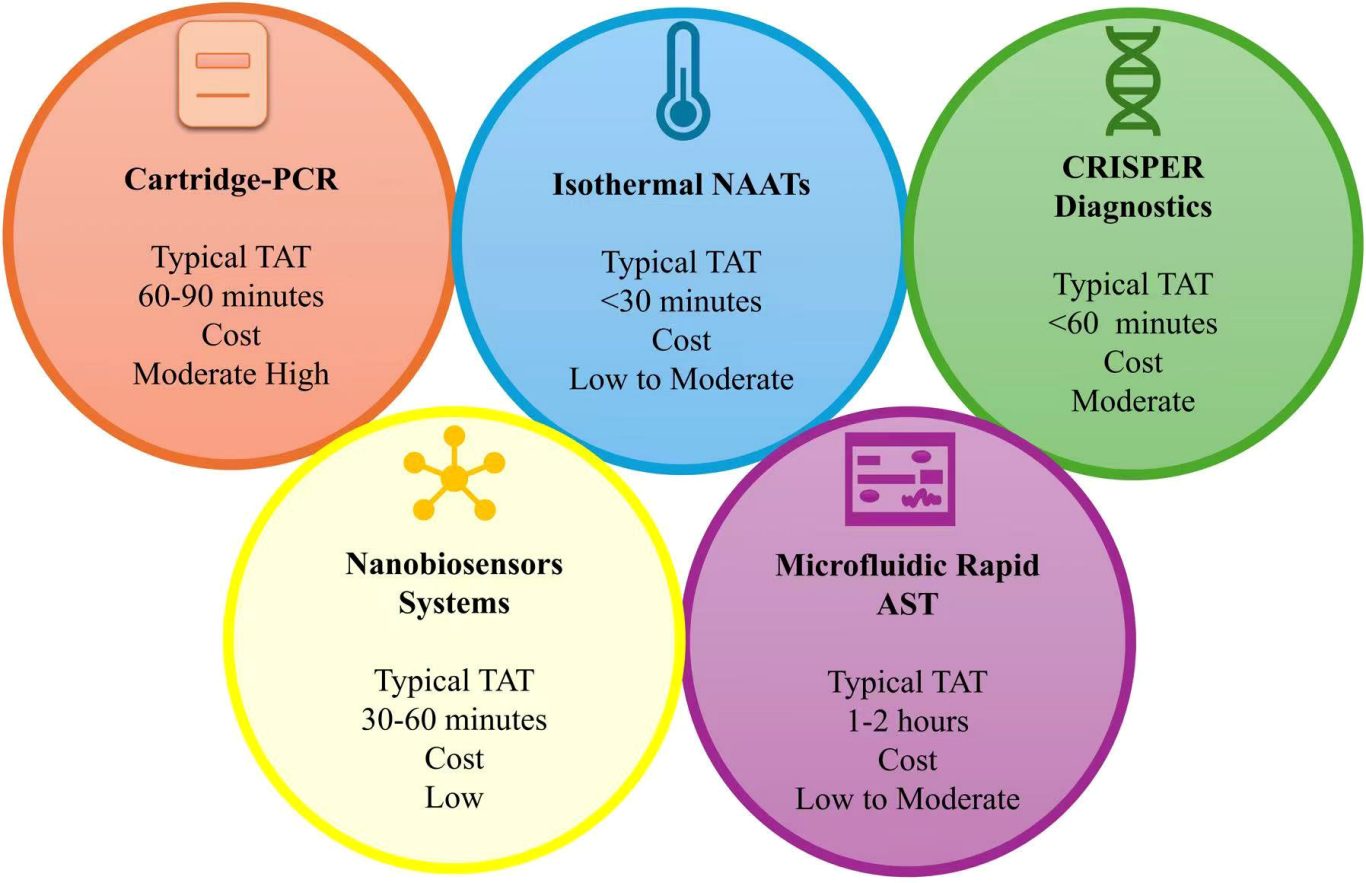
Comparative technological landscape of established and emerging molecular point-of-care platforms, illustrating typical turnaround times, cost tiers, ease of use, and suitability for drug-resistance detection. PCR, polymerase chain reaction; NAATs, nucleic acid amplification tests; CRISPR, clustered regularly interspaced short palindromic repeats; AST, antimicrobial susceptibility testing; TAT, turnaround time.

Collectively, POC molecular diagnostic platforms with integrated resistance detection have transitioned from experimental tools to realistic clinical options for managing high-priority infectious threats. Compared with purely phenotypic or antigen-based rapid tests, these molecular systems provide mechanistic insight into resistance, enabling earlier and more precise antimicrobial interventions (Alatawi et al., 2025). Deployment reduces diagnostic delays, informs therapy selection, strengthens antimicrobial stewardship, improves AMR surveillance, and opens avenues for future therapeutic strategies in neglected diseases. As summarized in **Table 1** and illustrated in **Figure 3**, each platform class offers distinct strengths and limitations, suggesting that no single technology will meet all diagnostic needs. However, inappropriate reliance on limited resistance panels, false-negative results, or misinterpretation of molecular resistance markers at the point of care may lead to suboptimal therapy selection and unintended antimicrobial exposure.

**Table 1:** Overview of established point-of-care molecular platforms for infectious-disease diagnosis and resistance detection.

Platform type	Example systems	Target pathogens	Resistance markers detected	TAT (time to result)	Sensitivity/specificity	Deployment notes
Cartridge-Based NAAT (PCR)	Cepheid Xpert MTB/XDR, Xpert Xpress, Abbott ID NOW	TB, SARS-CoV-2, Influenza, STIs	*rpoB*, *katG*, *inhA*, gyrA, macrolide resistance genes	30–90 min	90–98%/95–99% (varies by pathogen)	Widely deployed; CLIA-waived; robust sample-to-answer workflow
Isothermal Amplification	LAMP, RPA handheld systems	TB, HIV, HPV, emerging pathogens	β-lactamase genes, mecA, ESBLs	15–30 min	85–98%/92–100%	Low-power, portable, suitable for LMICs
CRISPR-Based POC	DETECTR, SHERLOCK prototypes	SARS-CoV-2, Dengue, TB, STIs	gyrA, *bla* variants, macrolide resistance SNPs	20–40 min	>98% specificity	High analytical precision; rapid reprogramming capability
Biosensor-Integrated Nano-POC	Graphene FETs, AuNP-LFA hybrids, electrochemical chips	Bacterial & viral pathogens	Plasmid-borne *bla*, gyrA SNPs, vanA/B	<15 min	High analytical sensitivity (pM–fM LOD)	Prototype stage; ideal for low-resource settings
Multiplex PCR POC	FilmArray-type panels	Respiratory pathogens, sepsis, STIs	mecA, *bla_KPC*, *bla_NDM*, vanA/B	45–90 min	92–99%	Syndromic testing; higher cost per test

These risks underscore the need for careful clinical integration, confirmatory testing pathways, and stewardship oversight to ensure that POC resistance diagnostics augment rather than undermine rational antimicrobial use. A tiered strategy combining cartridge-based NAATs, CRISPR assays, biosensors, and multiplex panels will likely define future POC resistance testing (Ghouneimy et al., 2022). Continued investment in clinical validation, regulatory harmonization, and integration with digital health infrastructure will be essential to fully realize the transformative potential of these technologies in combating antimicrobial resistance.

## 4 Emerging & future approaches for POC resistance diagnostics

The next generation of point-of-care (POC) resistance diagnostics is being shaped by the need to overcome the intrinsic limitations of current molecular platforms, including restricted target panels, inability to capture phenotypic resistance, and limited adaptability to rapidly evolving antimicrobial resistance (AMR) mechanisms. Emerging approaches aim to deliver faster, more comprehensive, and more flexible resistance profiling while maintaining operational simplicity suitable for decentralized and resource-limited settings. These innovations are particularly relevant for neglected infectious diseases such as tuberculosis, malaria, leishmaniasis, and multidrug-resistant bacterial infections. By integrating genotypic and phenotypic insights with digital connectivity, next-generation POC diagnostics promise to accelerate therapy decisions and guide precision interventions (Kaprou et al., 2021; Yamin et al., 2023).

### 4.1 Biosensor-based genotypic resistance detection

Biosensor-based genotypic resistance diagnostics are gaining increasing attention as a promising alternative to amplification-dependent molecular assays. These systems integrate highly specific molecular probes such as oligonucleotides, peptide nucleic acids, or CRISPR-derived recognition element with ultra-sensitive nanomaterial-based transducers, including graphene field-effect transistors (GFETs), plasmonic gold nanoparticles, carbon nanotubes, and electrochemical electrodes (Bakht et al., 2026). Signal changes upon target binding enable direct detection of resistance genes with exceptional sensitivity, sometimes at femtomolar or single-molecule levels (Huang et al., 2021). A key advantage of biosensor-based approaches lies in their exceptional analytical sensitivity, with some platforms capable of detecting target molecules at femtomolar or even single-molecule levels without extensive sample amplification. This capability opens the possibility of near-instantaneous AMR profiling at the point of care, significantly shortening diagnostic turnaround times. Low power requirements, miniaturization, and compatibility with portable devices make these platforms well suited for deployment in low-resource and outbreak-prone settings (Zhang et al., 2024). Furthermore, the low power requirements, miniaturization potential, and compatibility with portable readout devices make biosensor platforms well suited for deployment in low-resource, field-based, or outbreak-response settings. As fabrication costs decrease and multiplexing improves, biosensor-based diagnostics may support decentralized AMR surveillance and precision-guided therapy in neglected infectious diseases (Diouf and Savane, 2026).

### 4.2 Isothermal amplification and portable molecular workflows

Isothermal nucleic acid amplification technologies are playing an increasingly central role in the evolution of portable POC resistance diagnostics. Techniques such as loop-mediated isothermal amplification (LAMP), recombinase polymerase amplification (RPA), and rolling-circle amplification enable rapid target amplification at a constant temperature, eliminating the need for bulky and energy-intensive thermocyclers. These methods are now being integrated into compact, hand-held, or battery-operated devices that incorporate preloaded, lyophilized reagents and simplified sample processing workflows (Panno et al., 2020). They allow rapid detection of resistance genes in neglected infectious diseases, enabling informed therapy decisions in settings where empirical treatment has dominated. From an AMR perspective, these systems can be designed to rapidly screen for resistance-associated genes, allowing clinicians to make informed treatment decisions in settings where empirical therapy has traditionally dominated. Ongoing improvements in assay specificity, contamination control, and multiplexing capacity are further enhancing the clinical reliability of isothermal POC diagnostics. Their maturation will expand access to precision antimicrobial therapy and support timely interventions in high-burden, low-resource environments (Srivastava and Prasad, 2023).

### 4.3 Phenotypic rapid susceptibility testing (AST) at POC

Genotypic diagnostics are limited to known resistance mechanisms. Rapid phenotypic antimicrobial susceptibility testing (AST) at the POC provides culture-free evaluation of drug response, complementing genotypic assays. Advances in microfluidics, nanofluidics, and droplet-based technologies now enable the isolation and interrogation of individual bacterial cells within highly controlled microenvironments (Hattab et al., 2024). These platforms expose bacteria to defined concentrations of antimicrobial agents and monitor growth dynamics, metabolic activity, or cellular responses using optical, electrical, or biochemical readouts. Experimental POC phenotypic AST systems have demonstrated the ability to generate minimum inhibitory concentration (MIC)-equivalent results within 30 to 120 minutes, orders of magnitude faster than conventional culture-based susceptibility testing (Reszetnik et al., 2024). This approach is especially valuable for emerging resistance phenotypes, combination therapies, and infections where resistance mechanisms are heterogeneous or continuously evolving.

### 4.4 Genotypic databases, bioinformatics & adaptive target panels

The expanding global infrastructure for genomic surveillance of infectious pathogens provides a powerful foundation for the next wave of adaptive POC resistance diagnostics. Large-scale genotypic databases capturing resistance mutations, mobile genetic elements, and strain-level epidemiology can inform the dynamic design of diagnostic target panels tailored to regional and temporal AMR patterns. Cloud-connected POC platforms can be updated in near real-time, ensuring relevance as resistance landscapes evolve (Baker et al., 2023). By integrating genotypic precision, phenotypic relevance, and digital intelligence, next-generation POC diagnostics can bridge the gap between rapid testing and clinically actionable therapy. These emerging technologies signal a shift toward more comprehensive, flexible, and future-proof POC resistance diagnostics. By integrating genotypic precision, phenotypic relevance, and digital intelligence, next-generation POC systems have the potential to close the long-standing gap between rapid testing and clinically actionable susceptibility information. Successful translation will depend on rigorous clinical validation, regulatory harmonization, and thoughtful integration into existing healthcare and surveillance infrastructures. These emerging technologies promise to guide precision antimicrobial therapy, strengthen stewardship, and mitigate AMR in neglected infectious diseases.

## 5 Implementation challenges & barriers

Despite rapid advances in POC molecular technologies for AMR detection, multiple nontechnical barriers continue to constrain their widespread clinical and public health impact. These challenges span the entire diagnostic value chain, from sample acquisition and assay economics to regulatory oversight, manufacturing scalability, and health-system integration. Addressing these real-world constraints is essential to ensure that POC resistance diagnostics translate from technological promise into sustainable clinical practice (Shanmugakani et al., 2020). One persistent bottleneck is sample preprocessing. Most POC molecular platforms require high-quality DNA or RNA as input, yet clinical specimens such as sputum, stool, whole blood, and urine are inherently heterogeneous and frequently contain inhibitors that compromise assay performance. Effective pathogen lysis and nucleic acid extraction often remain complex, multi-step processes that are difficult to miniaturize or automate without sacrificing sensitivity or reproducibility. The lack of universally robust, rapid, and equipment-free sample preparation modules represents a critical vulnerability in current POC workflows and remains an area of unmet need for innovation (Niemz et al., 2011). Without reliable front-end processing, even highly sensitive downstream molecular assays fail to deliver consistent clinical value.

Economic considerations further limit adoption, particularly in low- and middle-income countries. Cartridge-based nucleic acid amplification tests and CRISPR-based diagnostics often rely on proprietary consumables, single-use cartridges, and cold-chain–dependent reagents. On a per-test basis, these molecular assays may cost 10–20 times more than conventional rapid antigen tests, creating significant financial barriers to large-scale deployment. The cumulative costs associated with consumables, instrument maintenance, and supply logistics exacerbate affordability challenges, especially in decentralized healthcare systems. Per-test costs may be 10–20 times higher than conventional rapid antigen tests, restricting deployment in resource-limited settings, including regions heavily burdened by neglected infectious diseases (Kim et al., 2025). Regulatory and quality assurance gaps present an additional obstacle, particularly for emerging diagnostic modalities such as biosensor-based or nanomaterial-enabled platforms. Demonstrating consistent analytical and clinical performance across diverse populations, specimen types, and geographic regions remains challenging, especially when pathogens exhibit substantial genetic diversity. Regulatory bodies, including the FDA, the European IVDR framework, and the World Health Organization, are still developing standardized evaluation pathways for these novel technologies. The absence of harmonized regulatory guidance can delay approval, increase development costs, and create uncertainty for manufacturers and healthcare providers alike (Kardjadj, 2025).

Manufacturing scalability and supply-chain robustness also pose major challenges. Many promising POC resistance diagnostics remain at the prototype or early commercialization stage, where scaling production from laboratory batches to millions of units introduces risks related to batch-to-batch variability, quality control, and long-term stability. Microfluidic devices and nanomaterial-based sensors are particularly sensitive to manufacturing inconsistencies, environmental conditions, and storage constraints. Ensuring reproducibility, extended shelf-life, and performance stability across diverse climatic settings is essential for global deployment but remains technically and logistically demanding (Cong and Zhang, 2022). Beyond technology and logistics, successful implementation depends heavily on healthcare system readiness. Integrating POC resistance diagnostics into clinical workflows requires updated treatment protocols, strong antimicrobial stewardship leadership, clinician training, and interoperable data-reporting systems. Without mechanisms for quality control, result interpretation, and linkage to therapeutic decision-making, even sophisticated diagnostics risk underutilization or misuse. The absence of integrated data infrastructures further limits the ability to leverage POC diagnostics for surveillance, outbreak detection, and policy-level AMR monitoring (Yoon et al., 2021). Finally, equity and access considerations must be addressed explicitly. There is a significant risk that advanced POC molecular diagnostics will preferentially benefit urban, tertiary-care, or well-resourced centers, thereby widening existing global health disparities. Ensuring equitable impact will require deliberate strategies focused on affordability, decentralized deployment, local manufacturing capacity, and accessible maintenance and training frameworks. Without such measures, technological innovation may inadvertently reinforce inequities rather than alleviate them (Aborode et al., 2025). These real-world constraints highlight that technological innovation alone is not sufficient. Successful translation demands coordinated policy, manufacturing, training, and health-system investment as explained in **Table 2**.

**Table 2:** Technical, economic, and regulatory barriers to POC diagnostic implementation and potential solutions.

Barrier type	Description	Impact	Proposed solutions
Sample Processing	Need for lysis, inhibitor removal, nucleic-acid prep	Limits decentralized use	Integrated microfluidic prep; reagent-free lysis; magnetic-bead extraction
Cost of Tests	High cartridge and consumable cost	Restricts LMIC adoption	Subsidization, pooled procurement, local manufacturing
Regulatory Uncertainty	Novel biosensor/CRISPR platforms lack clear pathways	Slows commercialization	Harmonized FDA–IVDR–WHO guidelines
Manufacturing Scalability	Micro/nanofabrication challenges	Batch variability & limited supply	Standardized fabrication; quality control automation
User Training & Workflow Integration	Inexperienced staff, lack of stewardship programs	Misinterpretation & underuse	Training modules, smartphone-guided workflows
Data Connectivity	Lack of cloud reporting & surveillance integration	Missed outbreaks, poor AMR tracking	API-based interoperability; national reporting systems

## 6 Future directions & therapeutic opportunities

**Figure 4:**
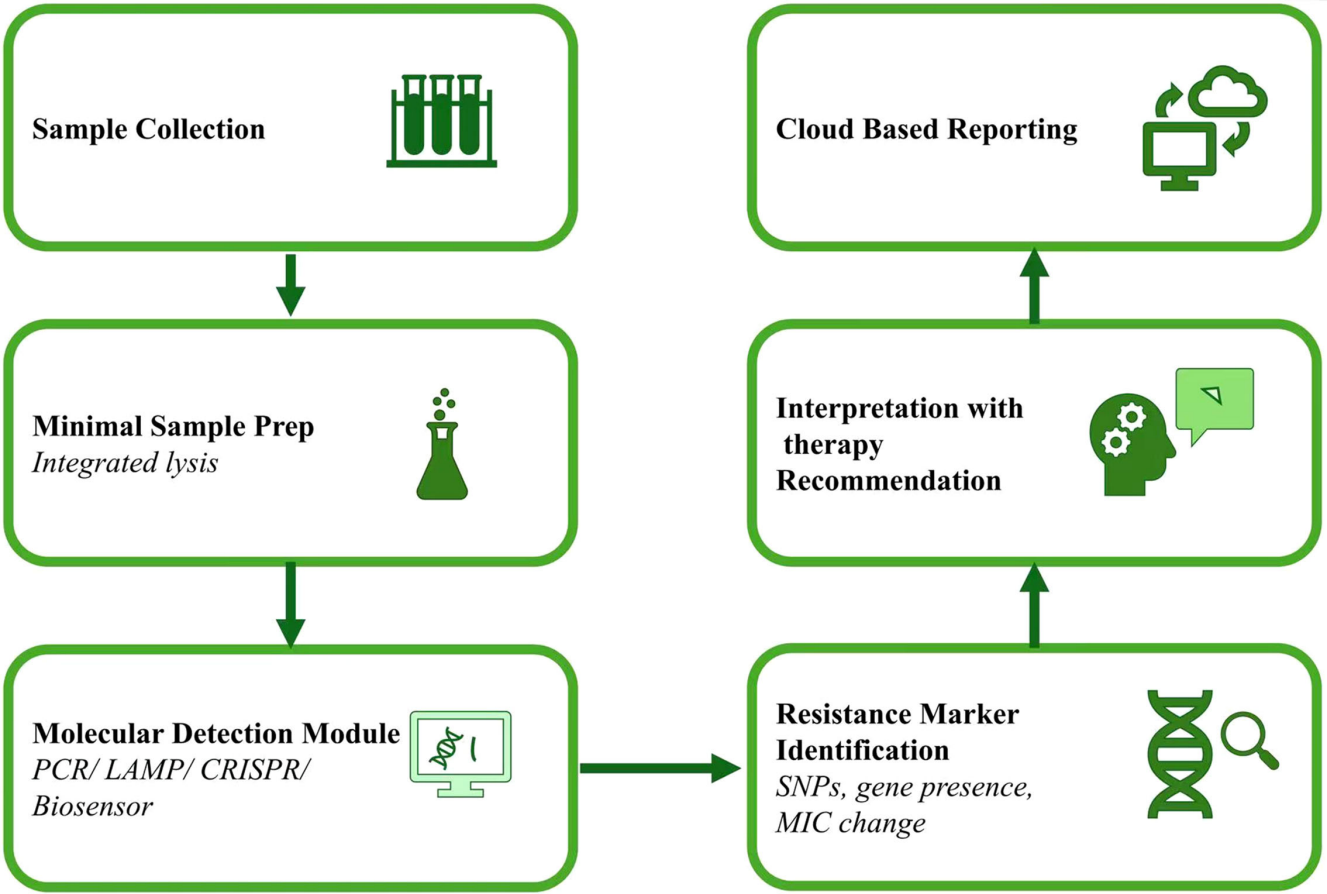
General workflow for point-of-care molecular resistance diagnostics, from sample acquisition and minimal sample preparation to molecular detection, resistance-marker identification, automated interpretation, and cloud-enabled therapeutic guidance. MIC change, Increase in the minimum drug concentration required to inhibit pathogen growth due to resistance-associated genetic or phenotypic alterations.

Molecular diagnostics, nanotechnology, microfluidics, and digital health platforms are converging to create a new era of managing infectious diseases, providing unprecedented opportunities for therapy as illustrated in **Figure 4**; **Table 3**. These advances are particularly transformative for neglected infectious diseases such as TB, malaria, leishmaniasis, and MDR bacterial infections.

**Table 3:** Integration of emerging POC diagnostic technologies with future therapeutic opportunities.

Diagnostic advancement	Mechanistic benefit	Therapeutic impact	Example future application
CRISPR-Adaptive Panels	SNP-level mutation identification	Precision antimicrobial selection	Rapid detection of novel carbapenemase variants
Microfluidic Rapid AST	Phenotypic MIC determination within 30–120 min	Saves critical time in sepsis management	ED rapid AST for Gram-negative bacteremia
Nanobiosensor POC Chips	Ultra-low LOD for pathogen/resistance gene detection	Early targeted therapy; outbreak control	Community screening for MDR-TB
Portable AI-Integrated POC	Automated interpretation; QC	Decreased errors; decentralization	Smartphone-integrated STI resistance testing
Cloud-Linked POC Surveillance	Real-time AMR trend monitoring	Data-driven stewardship	Digital AMR heatmaps for clinicians

### 6.1 Precision therapy guided by rapid resistance profiling

POC Diagnostic testing provides detection of antibiotic resistance in real-time, allowing clinicians to initiate targeted therapy during their first patient encounter, reducing the use of broad-spectrum antibiotics as empirical therapy and decreasing toxicity and reduction of the development of antibiotic-resistant infections (Alatawi et al., 2025).

### 6.2 Antimicrobial stewardship & outbreak forecasting

By incorporating POC diagnostics with cloud-based monitoring and AI based analytics, there is a capability for real time tracking of resistance patterns, prediction of outbreaks, and implementing data-driven antimicrobial stewardship interventions. This could significantly curb the spread of MDR and XDR strains, especially in high-burden regions (Pennisi et al., 2025).

### 6.3 Decentralized care in low-resource settings

The compact, battery-powered, and instrument-free system delivers relevant diagnostic and antibiotic resistance results in isolated, rural or conflict-stricken areas, enabling timely treatment and reduced mortality and transmission rates (Alatawi et al., 2025).

### 6.4 Rapid adaptation to emerging pathogens

Rapid adaptation to newly emerging or re-emerging pathogens will be achieved easily with the modular design features of CRISPR, isothermal, and biosensor-based systems, which provide the ability to quickly reconfigure. Such agility is critical in pandemic preparedness and response (Zhou et al., 2025).

### 6.5 Integration with novel therapeutics

The use of new antimicrobial modalities such as phage therapy, CRISPR-based antimicrobials, and targeted small molecules is increasing and POC resistance diagnostics would provide information that would help guide the rational use of these antimicrobial modalities, the monitoring of their therapeutic efficacy over time and the minimization of the development of resistance against these new modalities of treatment (Olawade et al., 2024).

### 6.6 Host–pathogen–resistance profiling

Future POC systems may integrate information related to host responses including inflammatory and immune responses as well as pathogen detection and resistance profiling to enable the ability to develop a personalised management approach for the infection developed based on the specific genotype of the pathogen, combined with an assessment of the host’s clinical condition (Lakshmanan and Liu, 2025). Overall, the integration of the above advances will lead us away from reactive management of infections to a more precise, data-intensive, and anticipation-based management of infections. This will benefit individual patients as well as the public health system overall.

## 7 Conclusions

Advancements in point-of-care (POC) molecular testing, including integrated drug resistance testing, will change how neglected infectious diseases such as TB, malaria, leishmaniasis and multi drug resistant bacteria are treated. Isothermal amplification, CRISPR technology, nanotechnology enhanced biosensors, microfluidics and AI have improved the sensitivity, ability to test multiple samples and ease of access to pathogen identification and drug resistance profiling by point-of-care providers; therefore, providers can diagnose, and treat patients more quickly, increase the ability to conduct surveillance and improve the antimicrobial stewardship of providers. However, there are still challenges associated with standardizing assays/homogenizing manufacturing through supply chain issues and ensuring equitable allocation of the tests. Next generation POC test systems will provide patient-specific precision therapy and help integrate with new direct acting therapeutics as well as support the implementation of proactive and data-driven disease management strategies, thereby enhancing preparedness for new epidemic threats and providing a more robust global response capability to the antimicrobial resistance crisis.
